# Integrative genomic analysis identifies epigenetic marks that mediate genetic risk for epithelial ovarian cancer

**DOI:** 10.1186/1755-8794-7-8

**Published:** 2014-01-30

**Authors:** Devin C Koestler, Prabhakar Chalise, Mine S Cicek, Julie M Cunningham, Sebastian Armasu, Melissa C Larson, Jeremy Chien, Matthew Block, Kimberly R Kalli, Thomas A Sellers, Brooke L Fridley, Ellen L Goode

**Affiliations:** 1 Department of Biostatistics, University of Kansas Medical Center, 3901 Rainbow Blvd, Kansas City, KS 66160, USA; 2 Department of Cancer Biology, University of Kansas Medical Center, Kansas City, KS 66160, USA; 3 Department of Health Sciences Research, Mayo Clinic, Rochester, MN 55905, USA; 4 Laboratory Medicine and Pathology, Mayo Clinic, Rochester, MN 55905, USA; 5 Medical Oncology, Mayo Clinic, Rochester, MN 55905, USA; 6 Office of the Director, Moffitt Cancer Center, Tampa, FL 33612, USA

**Keywords:** Integrative genomics, Ovarian cancer, Blood-based DNA methylation

## Abstract

**Background:**

Both genetic and epigenetic factors influence the development and progression of epithelial ovarian cancer (EOC). However, there is an incomplete understanding of the interrelationship between these factors and the extent to which they interact to impact disease risk. In the present study, we aimed to gain insight into this relationship by identifying DNA methylation marks that are candidate mediators of ovarian cancer genetic risk.

**Methods:**

We used 214 cases and 214 age-matched controls from the Mayo Clinic Ovarian Cancer Study. Pretreatment, blood-derived DNA was profiled for genome-wide methylation (Illumina Infinium HumanMethylation27 BeadArray) and single nucleotide polymorphisms (SNPs, Illumina Infinium HD Human610-Quad BeadArray). The Causal Inference Test (CIT) was implemented to distinguish CpG sites that mediate genetic risk, from those that are consequential or independently acted on by genotype.

**Results:**

Controlling for the estimated distribution of immune cells and other key covariates, our initial epigenome-wide association analysis revealed 1,993 significantly differentially methylated CpGs that between cases and controls (FDR, *q* < 0.05). The relationship between methylation and case-control status for these 1,993 CpGs was found to be highly consistent with the results of previously published, independent study that consisted of peripheral blood DNA methylation signatures in 131 pretreatment cases and 274 controls. Implementation of the CIT test revealed 17 CpG/SNP pairs, comprising 13 unique CpGs and 17 unique SNPs, which represent potential methylation-mediated relationships between genotype and EOC risk. Of these 13 CpGs, several are associated with immune related genes and genes that have been previously shown to exhibit altered expression in the context of cancer.

**Conclusions:**

These findings provide additional insight into EOC etiology and may serve as novel biomarkers for EOC susceptibility.

## Background

Epithelial ovarian cancer (EOC) is the fifth leading cause of cancer death among women in the United States and the most deadly among gynecologic malignancies. In 2013 it is estimated that 22,240 new cases of EOC will be diagnosed [1], making it one of the most common gynecologic malignancies. Along with the physical burdens suffered by affected patients, the costs to the health care system are significant [2] with recent estimates suggesting that EOC accounts for upward of 5.1 billion dollars annually; rendering this disease as one of the most expensive cancers to treat [3]. The enormous physical, societal, and economic burdens associated with EOC along with the current lack of success in the early diagnosis of this disease, underscore the urgent need of studies aimed toward understanding the molecular basis of EOC susceptibility.

Epigenetics refers to differences in phenotypic states that are not based on differences in the underlying DNA sequence, are potentially reversible, and are generally stably maintained during cell division. Epigenetic marks such as DNA methylation (DNAm) of cytosine residues in the context of CpG dinucleotides, have been extensively characterized in EOC tumor tissue and have been shown to differ between histological subtypes of ovarian cancer [4], associate with patient clinical outcomes including survival time [5] and progression [6], and have led to identification of inherited variants in *HNF1B* (hepatocyte nuclear factor 1 homeobox B) as a subtype-specific susceptibility gene [7]. Despite the obvious relevance of investigating tumor-derived DNAm signatures for understanding EOC risk and prognosis, it also is clear that tumors do not develop as isolated phenomenon in their target tissue, but instead result from altered processes affecting neighboring cells and tissues, including the immune system. Thus, alterations DNAm profiles measured in peripheral blood may be useful not only in understanding the carcinogenic process and response to environmental insults, but may also provide critical insights in a systems biological view of tumorigenesis. Recent work has begun to translate these findings to clinically useful endpoints by examining the relationship between DNAm alterations and cancer risk [8-11], including ovarian cancer [12]. Yet, the retrospective nature of such studies and the assessment of DNAm peripheral blood leukocytes present significant challenges in the interpretation of the results; in particular, (a) the extent to which the identified methylation marks are consequential or are causal/mediators of disease risk and (b) potential for confounding due to heterogeneity in the underlying population of cells used for methylation assessment [13-15].

These challenges have served to motivate the application of novel analytical approaches for retrospective studies of DNA methylation that aim to distinguish epigenetic marks that are consequential or reflect alterations to the methylome driven by the tumor itself, from those that are causal or mediate tumor growth and development. In particular, a recent case-control study of rheumatoid arthritis, Liu et al., [16] utilized genotype data collected on the study subjects to identify methylation marks that fall along the casual pathway from genotype to disease status. As the potential for confounding due to cell heterogeneity represents a major bottleneck in the interpretation of blood-based studies of DNA methylation, the authors also applied a recently developed statistical methodology [13] for estimating the underlying distribution of cell types across each of the study samples, enabling them to control for the potential confounding effects of cell type heterogeneity. The overarching paradigm of this work is that genetically driven alterations in the pattern of DNAm of white blood cells can result in functional deficits in the normal functioning of immune system that modify disease susceptibility. Here, we speculated that these same might hold true for EOC risk; that genetically induced changes in the epigenetic landscape of white blood cells can alter susceptibility to EOC. Indeed, integrative genomics studies of other cancers, for example prostate cancer, indicate that the tumor epigenetic landscape is partly mediated by genetic differences, which may affect disease progression [17,18]. Additionally, Genome-wide association studies (GWAS) in the context of ovarian cancer have identified 11 common risk alleles [7,19-24], and six of these are located in homeobox gene clusters (*HOXA*, *HOXB*, and *HOXD*), homeobox-related genes (*HNF1B*), or genes expressed in early progenitor cells (*BNC2*, *TERT*) [20,25,26]; many developmental genes such as these are silenced by DNAm in differentiated cells and become aberrantly hypomethylated during tumorigenesis [26].

Given the well-established role of genetic variation and EOC risk and importance of examining DNA methylation in non target tissues, we attempted to leverage these findings along with the analytical framework applied in Lui et al. [16], with the goal of gaining better understanding the epigenetic basis of EOC susceptibility. Specifically, using blood-derived genome-wide epigenetic and genetic data collected on a total of 214 EOC cases and 214 controls enrolled in the Mayo Clinic Ovarian Cancer Study, we aimed to distinguish blood-based DNA methylation markers that are candidates for mediating EOC genetic risk.

## Methods

### Study population and sample preparation

This study consisted of 428 women of European ancestry (214 pre-treatment invasive epithelial ovarian cancer cases and 214 controls one-to-one matched with EOC cases on the basis age (within 1-year)) between the ages of 27 and 91 enrolled in the Mayo Clinic Ovarian Cancer Study [20]. Genomic DNA was isolated from whole blood collected at the time of enrollment, using PureGene DNA isolation reagents (Gentra Systems, Minneapolis, MN), re-suspended in TE buffer, and stored at -80°C. Samples were bar-coded with a unique subject identification number to ensure accurate and reliable sample processing and storage. Research protocols were approved by the Mayo Clinic Institutional Review Boards, and all participants provided written informed consent.

### Genotype data

Leukocyte-derived DNA was genotyped with the Illumina 610-quad Beadchip Array™ according to manufacturer’s protocol, at the Mayo Clinic Medical Genome Facility (Rochester, MN) by laboratory personnel blinded to case-control status. Detailed quality control (QC) procedures have been described elsewhere [20,27]. Briefly, Illumina’s Genome Studio™ software was used to perform automated genotype clustering and calling. Assays included duplicates and laboratory controls, which showed sample concordance of 99.93%, genotype call rate of 99.7%. SNPs were excluded with call rate <95%, MAF <0.05, Hardy-Weinberg Equilibrium (HWE) p-value < 10^-4^, or unresolved replicate errors, and samples were excluded with call rate <95%, ambiguous gender, or predicted less than 80% European ancestry. SNPnexus was used to annotate the genotyped variants [28-30].

### DNA methylation assays

Leukocyte-derived DNA was assayed and underwent QC procedures at the Mayo Clinic Molecular Genome Facility (Rochester, MN). Samples were assayed in two batches, hereafter referred to as Batch 1 (*n* = 132; 66 cases and matched control samples) and Batch 2 samples (*n* = 296; 148 cases and matched control samples). For each sample, 1 μg of genomic DNA was bisulfite modified (BSM) using the Zymo EZ96 DNA Methylation Kit (Zymo Research, Orange, CA) according to the manufacturer’s protocol. Epigenome-wide assessment of DNA methylation was carried out using the Illumina Infinium HumanMethylation27 BeadChip, which is capable of interrogating the methylation status >27,000 CpG loci across the genome. This assay uses bisulfite-treated DNA and two site-specific probes for each marker, which bind to the associated methylated and unmethylated sequences. The intensity of the methylated probe relative to the total probe intensity (sum of methylated and unmethylated probe intensities) represents the fractional level of methylation for that specific site within a sample. Centre d’Etudes du Polymorphisme Humain (CEPH) DNA, placental DNA (positive control) and whole genome amplified (WGA) DNA (negative control) were also included (n = 9, n = 12 and n = 8, respectively), as were technical replicates (n = 12). Briefly, fragmented DNA was hybridized to the BeadChips, which were then processed through a primer extension and an immunohistochemistry staining protocol to allow detection of a single-base extension reaction. Finally, BeadChips were coated and then imaged on an Illumina iScan. Analysis included control probes for assessing sample-independent and sample-dependent performance.

### Methylation data pre-processing and quality control

The methylation level of each CpG locus was calculated in GenomeStudio® Methylation module (v.1.9.0) by comparing the ratio of fluorescent signal from the methylated allele to the sum from the fluorescent signal from both methylated and unmethylated alleles and scored as *beta* values, ranging from 0 (unmethylated) to 1 (methylated). We first excluded probes that had an rsid, were located on the Y chromosome, or were positioned at a single nucleotide polymorphism (SNPs) (dbSNP build 137), as SNPs at the same site have the potential to confound methylation assessment. We also removed CpG loci that had high beta values in BSM negative controls (defined as exceeding four standard deviations of the mean) and those that were detected in <70% of samples (based on a detection p-value cut-off of 0.05). This left a total of 25,926 out 27,578 (94%) of probes that passed QC. The intra-class correlation coefficients, computed based on beta values among CEPH replicates and for duplicate samples, were >0.93, indicating a high degree of reproducibility in our array. In addition, samples were excluded if >25% of the probes for that sample had detection p-values that exceeded 1 × 10^-5^. Following QC, 428 samples remained for analyses; including 132 samples (66 cases and matched control samples) and 296 samples (148 cases and matched control samples) in Batch 1 and Batch 2, respectively.

Next, we assessed possible plate/Beadchip/batch effects visually and through principal component analyses (PCA) [31,32]. DNAm values were logit-transformed (i.e., log_2_(β/1- β)) as in previous studies to obtain the DNAm M-value for each CpG locus [33,34]. PCA represents a feature extraction technique where the methylation data is orthogonally transformed, such that the first principal component has the largest possible variance (accounts for maximal amount of variability in the methylation data), and each succeeding component, in turn has the next highest variance possible. PCA was applied to the methylation data for each batch separately (*n*_*1*_ = 138 and *n*_*2*_ = 296, for Batch 1 and Batch 2, respectively) and also to the combined methylation data for both Batches (*n* = 428). The resulting top principal components (those explaining the maximal proportion of variability in DNAm) were then examined in terms of their association with technical aspects concerning the array (i.e., plate/BeadChip), and batch for the principal components estimated from the combined methylation data from the Batch 1 and Batch 2 samples. As batch was observed to be a major determinant of variability in the combined DNAm data (Additional files 1 and 2), we adjusted for batch-effects by applying the ComBat normalization method [35] using the R-package ‘sva’. Combat is an empirical Bayes batch adjustment methodology that uses a location and scale adjustment for standardizing the mean and variability in methylation levels across batches. This methodology been shown to perform effectively and efficiently compared to competing batch/plate-adjustment methodologies [36,37] and has become an established preprocessing step for array-based DNA methylation data [38-40]. Following the application of Combat, principal components were computed from the batch-adjusted data and inspected to ensure that batch effects had been successfully attenuated. In addition, within each batch we observed plate-effects (data not shown). To remove variability in DNAm due to plate, we fit a linear model to the logit-transformed methylation values for each CpG locus and included a fixed effect term for plate. The logit-transformed locus means were then added back onto the unstandardized residuals derived from these models, before back transforming values on the logit-scale to a 0 to 1 scale.

### Technical validation of the methylation array data

As an orthogonal array validation, eight CpGs with a broad spectrum of percent methylation (range; 0.11-0.73) and variability (standard deviation; 0.11-0.15) were assessed using bisulfite pyrosequencing. Ninety-six samples were tested, including 45 cases and 45 controls, two samples each of WGA, BSM negative, and control samples (CpGenome™ Universal Methylated DNA; Millipore Corporation, Billerica, MA). Primers (Additional file 3) were designed using the Pyrosequencing Assay Design Software. Genomic DNA (20-30 ng) was PCR-amplified using primers, one of which was biotinylated. Briefly, the incorporated biotinylated amplicon was immobilized on streptavidin-coated beads used to purify and render the denatured, single stranded and biotinylated PCR product. Single stranded DNA was purified using the pyrosequencing vacuum workstation. The single-stranded product was annealed to 0.3 μM of the sequencing primer complementary to the single-stranded template and placed at 85°C for two minutes, then cooled to room temperature for five minutes. Pyrosequencing reactions were performed on Biotage PyroMark MD, and data were analyzed using PyroMD Software. Percent methylation was quantified as methylated C to unmethylated C ratio using the Pyro Q-CpG software, which provided automatic QC for each sample for completion of bisulfite conversion and estimates of non-converted DNA. The median Pearson correlation of methylation values between the array and pyrosequencing assays was 0.88 (Additional file 3), suggesting high concordance in the methylation array values and those generated from pyrosequencing.

### Cell mixture deconvolution analysis

Recent work has demonstrated substantial differences in the DNAm signature across different leukocyte subtypes [13-15] and also differences in white blood cell proportions by EOC case-control status [41-43]. As such, heterogeneity in the underlying distribution of white blood cell types is likely to be a key confounder when examining the association between DNAm and EOC status. Using the plate- and batch-adjusted methylation data, we employed a statistical methodology [13] for inferring changes in the distribution of leukocytes based on peripheral blood DNAm signatures, in combination with a previously obtained external reference data set consisting of methylation signatures from purified leukocyte samples (i.e., B cells, natural killer (NK) cells, CD8+ T lymphocytes, CD4+ T lymphocytes, monocytes, and granulocytes) [13,14]. In this approach data obtained from a target set comprised of DNA methylation profiles from a heterogeneous mixture of cell populations is assumed to be a high-dimensional multivariate surrogate for the underlying distribution of cell types. Houseman et al. [13], proposed a cell mixture deconvolution methodology – similar to regression calibration – that involves the projection of DNA methylation profiles from the target set onto a reference data set, which consists of the DNA methylation signatures for isolated leukocyte subtypes. Under certain constraints, the cell mixture deconvolution approach can be used to approximate the underlying distribution of cell proportions within the target data via constrained projection. Application of this method to our data allowed us to estimate the expected difference in cell type proportions between ovarian cases and controls, as well as to predict the proportion of the aforementioned leukocyte subtypes for each of the study samples. In addition, these methods allowed us to quantify the proportion of total and systematic variability in peripheral blood DNAm explained by estimated immune cell composition.

Although this method has been shown to produce accurate and reliable estimates of the underlying distribution of cell type [44], we additionally investigated the consistency of our results with an independent study population. Specifically, we compared our estimates of the expected difference in cell type proportions between ovarian cases and controls with the results reported in Houseman et al. [13]; which consisted of the application of the cell mixture methodology using blood-derived methylation data from *n* = 131 pretreatment EOC cases and *n* = 274 controls [12].

### Causal inference test (CIT)

**Figure 1:**
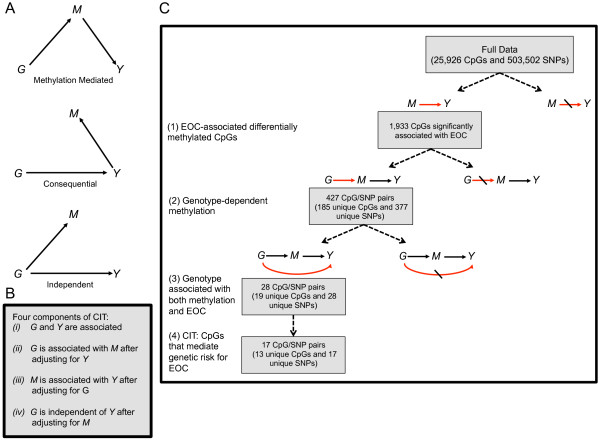
**Identification of epigenetically mediated genetic risk factors for EOC. (A)** Directed acyclic graphs (DAGs) depicting the possible relationships between a causal factor (G), a potential mediator (M), and an outcome (Y). Top, DAG for the methylation-mediated relationship, wherein G acts on Y through M. Middle, DAG for the methylation-consequential (reverse causality) relationship, in which changes in M arise as a consequence of Y. Bottom, DAG for the methylation-independent relationship, wherein G acts on M and Y independently. **(B)** The four components of the CIT. **(C)** Flow diagram illustrating the various filtering steps, and ensuing results, used to identify methylation sites that are candidates for mediators of genetic risk for EOC.

In a manner similar to that described in Liu et al. [16], genotype (G), methylation (M), and phenotype (Y) relationships were assessed using the causal inference test (CIT) [45] to classify them as “methylation mediated”, “methylation consequential” or “independent”. The CIT is comprised of a series of conditional correlation analyses that consider the possible directed relationships between a causal factor (genotype (G)), a potential mediator (methylation (M)) and an outcome (EOC status (Y)) (Figure 1A). In order for methylation (M) to be classified as a mediator of genetic (G) risk for EOC (Y) the following conditions must be met: (1) G and Y are associated, (2) G is associated with M after adjustment for Y, (3) M is associated with Y after adjusting for G, and (4) G is independent of Y after adjusting for M (Figure 1B). When M is a consequence of Y or independently acted on by G (Figure 1A), there should be no difference in the effect of G on Y, when conditioning on M. However, when M mediates the genetic risk for EOC, conditioning on M should substantially reduce the effect of G on Y [16,45].

The CIT P-value was defined using the intersection-union framework as the maximum of the component P-values for the first three of these conditions. G, M, and Y relationships were considered methylation-mediated if: the p-value obtained from the fourth condition above was > 0.05, indicating no statistically significant association between G and Y after adjustment for M; and the CIT P-value was < 0.05. Where appropriate, linear and logistic regression models were used to examine the four conditions comprising the CIT.

To ease the computational burden that would ensue from examining the above conditions for every G, M, and Y trio (503,502 × 25,926 × 4 total tests), we implemented the three step filtering procedure described in [16]. In the first step, the methylation status of each CpG, epigenome-wide, was examined for its association with Y. In step two, potential genetic regulators of methylation were identified by computing the pairwise relationship between each SNP, genome-wide, and each of CpG sites that were associated with Y in step 1. The final step involved an examination of the relationship between Y and each SNP that was identified as statistically significant in step 2. The general scheme of this analytic procedure is given in Figure 1C.

### Identifying ovarian cancer-associated differentially methylated CpGs

To discern differentially methylated CpGs between EOC cases and controls, we fit a series of linear regression models, which modeled the methylation M-value for each CpG as a function of ovarian case/control status. Models were adjusted for the estimated differential leukocyte cell counts described above, as well as age (continuous), current smoking status (yes vs. no), alcohol consumption (never, former, and current), study enrollment year (1999-2002, 2003, 2004, 2005, and 2006-2007), location of residence (MN vs. other), parity and age at first birth (nulliparous, 1-2 at ≤ 20 yrs, 1-2 at > 20 yrs, 3+ at ≤ 20 yrs, and 3+ > 20 yrs), and the first principal component representing within-European population sub-structure. Due to the large number of tests being performed, we corrected for multiple comparisons by computing the false discovery rate (FDR) q-value [46].

### Identifying genotype-dependent differentially methylated CpGs

All epigenome-wide statistically significant (FDR q-value < 0.05) ovarian cancer- associated differentially methylated CpGs were subsequently examined based on their association with genotype using an additive minor-allele dosage model fit to all of the study subjects. Briefly, we used a series of linear regression models (# ovarian cancer-associated CpGs × # of SNPs) that modeled methylation M-values, as a function of the number of minor alleles for a specific SNP. Genotype-methylation associations were adjusted for multiple comparisons by computing the FDR q-value. A less stringent FDR q-value cutoff of 0.10 was used to determine statistical significance, so as to limit false negative findings.

## Results

### Study population

The study population considered here consisted of 428 women of European ancestry (214 pre-treatment invasive EOC cases and 214 controls) between the ages of 27 and 91 enrolled in the Mayo Clinic Ovarian Cancer Study. Of the EOC cases (*n* = 214), 114 had tumors of serous histology (66%), 49 tumors were endometriod (23%), 13 were clear cell (6%), 5 were mucinous (2%) and 6 (3%) were other/unknown. Further information on clinical, lifestyle, and demographic characteristics of the study population is provided in Table 1 and Additional file 4. In general, baseline characteristics of EOC cases versus controls were similar to those estimated based on previous studies of known risk factors (Additional file 4).

**Table 1:** Clinical characteristics for the study population

	**Batch 1 (N = 132)**	**Batch 2 (N = 296)**	**Total (N = 428)**
**EOC status**			
Control	66 (50.0%)	148 (50.0%)	214 (50.0%)
Case	66 (50.0%)	148 (50.0%)	214 (50.0%)
**Age**			
Mean (SD)	60 (12)	63 (13)	62 (13)
Median	61	65	64
Q1, Q3	50, 69	54, 73	52, 72
Range	(33–82)	(27–91)	(27–91)
**Year enrolled**			
1999–2002	18 (13.6%)	164 (55.4%)	182 (42.5%)
2003	10 (7.6%)	36 (12.2%)	46 (10.7%)
2004	41 (31.1%)	33 (11.1%)	74 (17.3%)
2005	25 (18.9%)	23 (7.8%)	48 (11.2%)
2006–2007	38 (28.8%)	40 (13.5%)	78 (18.2%)
**Parity, number of births**			
Nulliparous	24 (18.2%)	42 (14.2%)	66 (15.4%)
1-2,<=20 yrs	7 (5.3%)	14 (4.7%)	21 (4.9%)
1-2,>20 yrs	31 (23.5%)	84 (28.4%)	115 (26.9%)
3+,<=20 yrs	28 (21.2%)	47 (15.9%)	75 (17.5%)
3+,>20 yrs	40 (30.3%)	95 (32.1%)	135 (31.5%)
**Smoking status (current)**			
No	120 (90.9%)	259 (87.5%)	379 (88.6%)
Yes	10 (7.6%)	19 (6.4%)	29 (6.8%)
**State**			
Other	68 (51.5%)	120 (40.5%)	188 (43.9%)
Minnesota	64 (48.5%)	176 (59.5%)	240 (56.1%)
**Alcohol consumption**			
Never	32 (24.2%)	71 (24.0%)	103 (24.1%)
Current	78 (59.1%)	152 (51.4%)	230 (53.7%)
Former	18 (13.6%)	47 (15.9%)	65 (15.2%)
**Histology**			
Serous	43 (65.2%)	98 (66.2%)	141 (65.9%)
Mucinous	1 (1.5%)	4 (2.7%)	5 (2.3%)
Endometrioid	16 (24.2%)	33 (22.3%)	49 (22.9%)
Clear Cell	4 (6.1%)	9 (6.1%)	13 (6.1%)
Other	2 (3.0%)	4 (2.7%)	6 (2.8%)
**Grade**			
Grade 1	1 (1.6%)	9 (6.1%)	10 (4.7%)
Grade 2	10 (15.6%)	21 (14.2%)	31 (14.6%)
Grade 3	32 (50.0%)	79 (53.4%)	111 (52.4%)
Grade 4	21 (32.8%)	39 (26.4%)	60 (28.3%)
**Stage**			
Stage 1	11 (16.7%)	29 (19.6%)	40 (18.7%)
Stage 2	3 (4.5%)	11 (7.4%)	14 (6.5%)
Stage 3	43 (65.2%)	84 (56.8%)	127 (59.3%)
Stage 4	9 (13.6%)	24 (16.2%)	33 (15.4%)

### Inferred immune cell subsets differ between ovarian cases and controls

**Figure 2:**
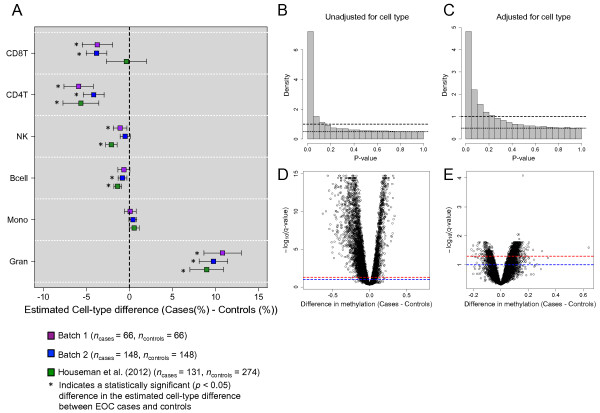
**Differential cell distributions in EOC cases. (A)** Estimated difference in leukocyte subtypes (i.e., CD8+ T-lymphocytes (CD8T), CD4+ T-lymphocytes (CD4T), natural killer cells (NK), B cells (Bcell), monocytes (Mono), and granulocytes (Gran)) between EOC cases and controls. Bars reflect the 95% confidence interval for the difference in cell distributions between EOC cases and controls. (B, C) Histograms of *P*-values obtained from examining the association between DNAm and EOC case/control status, **(B)** unadjusted for estimated cell distribution and **(C)** adjusted for the estimated cell distribution. Dashed line is the density histogram that is expected if all CpGs were null (not differentially methylated) and the dotted line is at the height of our estimate of the proportion of null p-values. **(D, E)** Volcano plots of –log_10_(*q*-value) against the estimated difference in methylation between EOC cases and controls, **(D)** unadjusted for estimated cell distribution and **(E)** adjusted for the estimated cell distribution. Red and blue dashed lines indicate –log_10_(*q* = 0.05) and –log_10_(*q* = 0.10), respectively. Each model was fit to the combined data from the Batch 1 and 2 samples (*n* = 428) and were adjusted for age, smoking status, alcohol consumption, study enrollment year, location of residence, parity, and population substructure.

As DNAm was profiled using genomic DNA from whole-blood, which is comprised of genetic substrate from various leukocyte subtypes, the methylation signatures in our study population represent the aggregate methylation profile across a complex cellular landscape. To examine the predicted differences in the major leukocyte components of whole blood (i.e., B cells, natural killer (NK) cells, CD8+ T lymphocytes, CD4+ T lymphocytes, monocytes, and granulocytes) we utilized the cell mixture methodology of Houseman et al. [13]. This method uses a reference panel consisting of the DNAm signatures of isolated leukocyte subtypes to deconvolve the distribution of white blood cell types when DNAm is profiled in whole blood. As noted in Figure 2A, several of the estimated cell type proportions showed statistically significant (*p* < 0.05) differences between EOC cases and controls. More importantly, the relationship between cell type and EOC status was highly consistent between both batches of samples within our study population and also with the results reported in a prior publication [13], which consisted of the application of the cell mixture methodology to an independent study population of *n* = 131 pretreatment EOC cases and *n* = 274 controls [12]. In particular, granulocyte fractions were higher in EOC cases, while CD8+ T lymphocyte and CD4+ T lymphocyte, and, to a lesser extent B cell and NK cell fractions, were lower in EOC cases compared to controls.

These results combined with known methylation differences by cell type suggest that it is critical and feasible to adjust for the underlying distribution of cell types when investigating the relationship between DNAm and EOC case/control status. In particular, Figure 2 shows the epigenome-wide association between DNAm and EOC status (Batch 1 and 2 samples combined) before (Figure 2B,D) and after (Figure 2C,E) adjustment for the estimated cell type proportions, and demonstrates a substantial reduction in the number of differentially methylated CpGs by EOC case/control status upon adjustment.

### Identifying CpG dinucleotides that mediate genetic risk for EOC

Case-control studies focused on the identification of patterns of differential DNAm in the context of disease phenotypes are limited by their retrospective nature and, therefore, are unable to discriminate between patterns that are a consequence of the disease and those that are mediators of disease risk. To filter out consequential epigenetic marks in an attempt to understand biology related to the cause of EOC, we adopted the framework described by Liu et al. [16] for identifying epigenetic marks that are candidate mediators of genetic risk for EOC. To identify instances in which genetic variation influences risk for EOC by regulating CpG-specific methylation patterns we performed a three-step filtering procedure followed by the Causal Inference Test (CIT) [45]. In the first filtering step, we conducted an epigenome-wide association study (EWAS) to identify CpGs differentially methylated by EOC case/control status. Using a series of linear regression models that were adjusted the estimated cell-type proportions and other key covariates, we found 1,993 out of 25,926 (7.7%) CpGs were associated with EOC case/control status after controlling for multiple comparisons (FDR, *q* < 0.05), step 1 Figure 1C, Additional file 5). As partial validation of these results, we examined the methylation of these 1,993 CpG loci in an independent study population [12]. Our validation analysis revealed that 1,603 out of 1,993 CpG loci (80%) were significantly differentially methylated (FDR; *q* < 0.05), and of these 1,603 loci, 94% exhibited the same direction of association (i.e., hyper versus hypomethylated) compared to the results obtained from our study population (Additional file 5). Similar to the models fit to our study population, models fit to the independent data were adjusted for the estimated distribution of cell types and subject age; however smoking status, alcohol consumption, parity and population substructure variables were not available in these data and therefore could not be used for adjustment.

**Figure 3:**
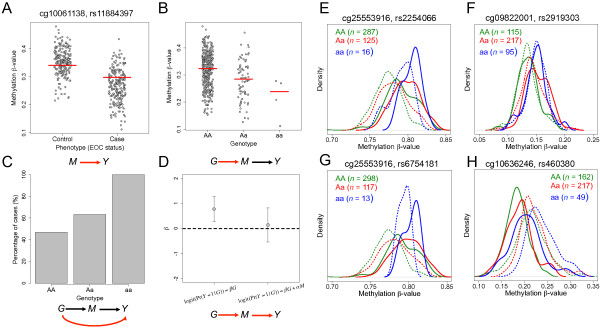
**Genotype-dependent candidate CpGs that mediate genetic risk for EOC.** (Left) Plot depicting the DNAm status of *cg10061138*, associated with gene *STAB1*, between **(A)** EOC cases and controls and by genotype at SNP *rs11884397***(B)**. Red lines denote the median methylation levels. **(C)** Percentage of EOC cases by the number of minor alleles for SNP *rs11884397*. **(D)** Coefficient (β) reflects the log-odds of EOC for a one-unit increase in the number of minor alleles for SNP *rs11884397* with and without adjustment for the methylation levels of *cg10061138*. Bars represent the 95% CI for the estimate of the log-odds (i.e., β). **(E-H)** Density plots of DNAm by genotype (AA = green, Aa = red, and aa = blue) for four EOC–associated CpGs; solid lines indicate the methylation distribution for EOC cases and dotted lines indicate the methylation distribution for controls.

In an attempt to identify CpGs where methylation might be genetically influenced, we performed a genome-wide SNP association analysis for each of the 1,993 CpG loci that were differentially methylated between EOC cases and controls. Fitting an allelic dosage model to each of these CpGs and each of 503,502 SNPs, we identified 427 CpG-SNP pairs with genome-wide statistical significance (FDR; *q* <0.10) (step 2 Figure 1C, Additional file 6). These 427 CpG-SNP pairs constituted 377 unique SNPs and 185 unique CpGs, and these CpG loci were disproportionately located in CpG islands (Fisher’s Exact; *p* = 0.017); CpG-dense regions present in the promoters of 50%–70% of human genes. Nonetheless, it is still possible that the differential patterns of methylation observed for these 185 CpGs are a consequence of EOC or independently acted on by genotype. To address this concern, we next examined the association between the 377 unique SNPs and EOC status. Of the 377 SNPs, we identified 28 that were significantly associated with EOC status at *P* < 0.05 (Additional file 7). These 28 SNPs form 28 CpG-SNP pairs with 19 unique CpGs (step 3 Figure 1C). Implementing the CIT test, we found that the SNP association with EOC was attenuated upon adjustment for methylation for 17 of the 28 CpG-SNP pairs (61%), suggesting mediation (Figure 3). These 17 CpG-SNP pairs constituted 13 unique CpGs and 17 unique SNPs and represent potential methylation-mediated relationships between genotype and EOC risk (step 4 Figure 1C, Table 2). Information regarding the genomic location and additional annotation for these CpGs and SNPs is provided in Additional files 8 and 9, respectively.

**Table 2:** CpG sites that were identified as potential mediators of genetic risk for EOC (CIT p < 0.05)

	**CpGs associated with EOC**				**SNPs associated with CpGs**						
**CpG**	**Meth diff**	**P-value (M vs Y)**	**Gene**	**Chr**	**Pos**	**SNP**	**Chr**	**Pos**	**P-value (G vs M)**	**P-value (G vs Y)**	**P-value (CIT)**
cg03718677	-0.08	4.60E-03	*TMOD4*	1	149414890	rs1250220	2	216320050	2.30E-07	0.023	0.023
cg03718677	-0.08	4.60E-03	*TMOD4*	1	149414890	rs1250252	2	216313591	1.70E-07	0.029	0.029
cg09822001	0.11	5.50E-04	*APOA1BP*	1	154827958	rs2919303	8	62214493	1.50E-07	0.027	0.027
cg10636246	-0.11	2.70E-04	*AIM2*	1	157313597	rs11120596	1	215850817	7.60E-07	0.002	0.002
cg10636246	-0.11	2.70E-04	*AIM2*	1	157313597	rs460380	21	46028864	5.30E-07	0.01	0.01
cg13721560	0.07	7.90E-03	*LRPPRC*	2	44076072	rs2289840	3	124181739	9.40E-08	0.041	0.041
cg10061138	-0.09	8.30E-04	*STAB1*	3	52504125	rs11884397	2	23885388	3.10E-07	0.002	0.002
cg25086702	0.1	1.70E-03	*HMGB2*	4	174492134	rs11210834	1	43787437	7.10E-08	0.048	0.048
cg24136586	0.1	5.50E-03	*ATG10*	5	81304122	rs3800524	6	168302015	1.80E-06	0.034	0.034
cg01495509	0.08	5.30E-03	*KCNMB1*	5	169748956	rs4457945	15	54608127	2.20E-08	0.019	0.019
cg25553916	0.06	6.90E-04	*FLJ22318*	5	177491361	rs2254066	2	29889019	2.60E-07	0.003	0.003
cg25553916	0.06	6.90E-04	*FLJ22318*	5	177491361	rs2631958	2	29958841	7.10E-07	0.009	0.009
cg25553916	0.06	6.90E-04	*FLJ22318*	5	177491361	rs6754181	2	29966380	3.10E-07	0.003	0.003
cg19436567	0.12	1.50E-04	*ARID1B*	6	157141067	rs12362925	11	16875419	7.80E-08	0.029	0.029
cg08142684	0.13	2.70E-04	*TCP1*	6	160129858	rs9792311	8	123940585	7.40E-07	0.023	0.023
cg05109049	0.09	5.30E-03	*EVI2B*	17	26665459	rs2294405	6	99292136	1.60E-06	0.013	0.013
cg00021527	0.09	5.20E-03	*TAF15*	17	31160293	rs10488500	7	76841259	2.50E-07	0.046	0.046

**M:** Methylation, **G:** Genotype, **Y:** EOC status.

**Methdiff:** Difference in the methylation M-value between EOC cases minus controls, adjusted for the estimated distribution of cell types, age, smoking status, alcohol consumption, study enrollment year, location of residence, parity and age at first birth, and the first principal component representing within-European population sub-structure.

**Gene:** Denotes the nearest gene for the given CpG site.

Examining the linkage-disequalibrium (LD) structure among the 17 unique SNPs identified in our analysis showed that most of the identified SNPs were uncorrelated at r^2^ < 0.05, with the exception of rs1250220 and rs1250252 located in an intragenic region on chromosome 2q35 and rs6754181, rs2631958, rs2254066, located in an intron region associated with *ALK* (anaplastic lymphoma receptor tyrosine kinase) (Additional file 10). The 17 CpG-SNP pairs highlight 13 CpG loci; all loci correlate with a single SNP or SNP cluster, although cg10636246 located near *AIM2* demonstrated an association with two independent SNPs with different genomic locations (rs11120596 (*p* = 8 × 10^-7^) and rs460380 (*p* = 5 × 10^-7^)) (Table 2).

As differences in tumor DNAm, epidemiologic risk factors, genetic variants, and precursor tissues are known to exist between the major EOC histologies (serous, mucinous, endometrioid, and clear cell) [4], we evaluated whether the 13 unique methylation mediators exhibited consistent patterns of methylation in a case-only analysis across EOC subtypes. With the exception of cg25553916 located in the promoter region of *FLJ22318* (required for meiotic nuclear division 5 homolog B), which showed increased methylation in mucinous cases (*p* = 0.006), the methylation levels of the 12 remaining CpG loci were not statistically significant different across the histologies of EOC (Additional file 11). Interestingly, this locus was the only one among the 13 that did not exhibit the same direction of association with EOC comparing our results to those from the Teschendorff et al. [12] data (Table 2 and Additional file 5).

## Discussion

Attempts aimed at distinguishing causal methylation marks from those that are merely a consequence of disease are critical for elucidating the biological mechanisms underlying this disease. Previous analyses of genetic regulators of methylation and expression levels have revealed three-way causal relationships, where the prevailing model is one in which genetic variation influences methylation that in turn influences expression levels. The idea that DNA methylation levels at specific loci are under genetic control has gained traction in recent years, bolstered by the results obtained from comparing patterns of DNA methylation between monozygotic and dizygotic twins [47]. Here, we aimed to leverage these findings in an attempt to filter out epigenetic marks resulting from disease, focusing our attention on the identification of epigenetic marks that are potential mediators of genetic risk for EOC. Not only are such analyses critical for our understanding of EOC pathogenesis, but the genotype-methylation markers identified through such efforts may further enhance the growing library of risk-associated biomarkers for EOC.

Associations between genetic variation with expression and methylation levels have been identified in several organisms [48,49] and tissue types [50]. While recent work has demonstrated both local (*cis*) and distal (*trans*) associations of genetic variation with methylation levels [51-53], little is known about the precise biological mechanisms by which genetic variants modify DNA methylation. All of the methylation-genotype pairs identified in our analysis indicated *trans* regulation, or distant regulation effects. Although none of the 17 SNPs identified in our analysis have been previously identified as reaching genome-wide statistical significance in GWAS of ovarian cancer, of particular importance was the identification of *ALK* as a potential regulator of CpG-specific DNAm and genetic risk marker for EOC. This gene encodes a receptor tyrosine kinase belonging to the insulin receptor superfamily, and has been found to be rearranged, mutated, or amplified in a series of human cancer tumorigenesis [54-56]. Recent work has demonstrated methylation induced silencing of IL-2Rγ expression in in T-cell lymphoma cells expressing NPM-ALK kinase [57], which originates from fusion of the nucleophosmin (NPM) and the membrane receptor anaplastic lymphoma kinase genes. IL-2Rγ is shared by receptors for several cytokines that play key roles in the maturation and growth of normal CD4+ T lymphocytes and other immune cells. Thus, it is possible that genetic variation in *ALK* contributes to epigenetic modifications that alter the normal functioning of immune cells, however the exact biological mechanisms by which *ALK* exerts an influence on DNA methylation is unclear.

It is also compelling that the predicted distribution of immune cell subsets in our data, which showed increased myeloid derived cell types (e.g., monocytes and granulocytes) and decreased lymphocytes (e.g., CD8+ T lymphocytes, CD4+ T lymphocytes, B cells, and natural killer cells) between EOC cases and controls, mimicked the results obtained when applying the cell mixture methodology to an independent study population. The relationship between predicted cell type distributions and cancer status are consistent with previous literature, where it has been demonstrated that EOC cases have decreased B and T-lymphocyte fractions [41-43] and increases in neutrophil granulocytes [43]. While modest variation was observed in the estimated cell type differences between EOC cases and controls between the different study populations, particularly for CD8+ T-lymphocytes, this is not entirely unexpected given differences in the distribution of ovarian cancer histological subtypes between the study populations (Table 1; Teschendorff et al. [12]) and that variation in host immune responses to EOC has been shown to vary by histological subtype [58].

Our analysis identified cg25086702 as a potential mediator of genetic risk for EOC. This particular locus resides in CpG island region located in *HMGB2* (high mobility group protein 2) and was found to be hypermethylated in EOC in the present analysis (β = 0.10, 95% CI [0.04, 0.16]). High mobility group box (*HMGB*) proteins are ubiquitous, abundant nuclear proteins with diverse functions in the cell. *HMGB1* and *HMGB2* are the main members of the *HMGB* protein family and their overexpression has been observed in numerous human malignancies, including hepatocellular [59], skin squamous cell [60], prostate [61], gastrointestinal [62,63] breast [64,65], and bladder carcinomas [66]. Additionally, a recent report demonstrated increased expression of *HMGB2* in invasive EOC tumors compared to EOC tumors with low malignancy potential [67]. However, many of the results demonstrating *HMGB2* overexpression were based on measurements derived from tumor tissue, and far less is known about the implications of dysregulated *HMGB2* expression in peripheral blood leukocytes and its role in cancer risk. While it is possible that our observation of increased CpG island methylation of *HMGB2* in EOC cases is due to the methylation signature arising from circulating tumor cells (CTCs), this is unlikely as CTCs would be expected to comprise a small fraction of the total cells used in assessing DNAm [68], and thus contribute insignificantly to the overall methylation signatures analyzed. Also, given the role CpG island hypermethylation on gene silencing and the numerous reports of *HMGB* overexpression in tumor tissue, we might expect to see the opposite results (i.e., CpG island hypomethlation of *HMGB2*) if in fact, CTCs were driving force behind the methylation signals detected here. An alternative explanation for these findings is motivated by the role of *HMGB1*, a closely related gene, in immune response in adult peripheral blood. *HMGB1* recruits inflammatory cells and activates innate immune cells. Furthermore, after being secreted by activated macrophages or its release from necrotic cells, *HMGB1* regulates adaptive immunity [69-71]. Thus, CpG island hypermethylation induced silencing of *HMGB1* and possibly other *HMGB* genes, may compromise the immune system, promoting tumor development and progression.

Our analysis also identified cg05109049 (β = 0.09, 95% CI [0.03, 0.16]), associated with *EVI2B (*Ecotropic Viral Integration Site 2B Protein), as a potential mediator of genetic risk for EOC. *EVI2B* is expressed in peripheral blood mononuclear cells, fibroblasts, and bone marrow and blood-based overexpression of this gene was recently reported in postoperative relapse of colorectal cancer [72]. Another notable discovery from our analysis was hypermethylation of cg00021527 (β = 0.09, 95% CI [0.03, 0.15]), residing in a CpG Island region located in the gene *TAF15* (TATA box-binding protein-associated factor 2 N 68 kDa), which together with FUS (fused in sarcoma) and EWS (Ewing sarcoma breakpoint 1), constitute the FET protein family. The FET-proteins are involved in transcriptional regulation and RNA processing, and FET-gene deregulation is associated with development of cancer. In particular, a recent report demonstrated that *TAF15* knockdown affects the expression of a large subset of genes, including many involved in cell cycle and cell death [73]. Together, these findings highlight the biological relevance of the methylation sites identified in our investigation and their potential role in the pathogenesis of EOC.

There are several noteworthy limitations to the present study. First, the relatively small sample size and large number of genotype/methylation markers, reduces our statistical power for detecting genotype/methylation associations. To address the burden of multiple comparisons arising from the large number of genotype/methylation markers, we employed an analytical strategy that is based on a series of filtering steps, resulting in many fewer overall tests than an analysis considering all possible genotype, methylation, and phenotype combinations. Further, while Illumina Infinium HumanMethylation27 BeadChip provides an efficient solution for surveying genome-wide DNA methylation profiles, the lower coverage and scope of this array compared to more recent array technologies, e.g., Illumina Infinium HumanMethylation450 BeadChip, may have limited our ability for detecting methylation mediators of EOC genetic risk.

With regard to our sample size, efforts to replicate the analysis described here using a larger group of study subjects as both a validation of our existing results, and to identify additional methylation sites that mediate genetic risk for EOC is ongoing research by our group. Moreover, we additionally evaluated the results of our EWAS analysis using a previously published data set, which consisted of whole-blood derived methylation data – assayed using the same array technology – collected from pre-treatment EOC cases and controls. Unlike our data, genotypic information was not available on those subjects, preventing a complete validation of the genotype-methylation pairs identified here. However, the fact that 12 of the 13 (92%) CpGs representing potential methylation-mediated relationships between genotype and EOC risk demonstrated the same direction of association with EOC status is encouraging and serves as motivation for the continued and future study of these markers.

A second consideration of this work involves the potential for confounding based on interpersonal variability the distribution of cell types used in assessing DNA methylation. While previous reports involving blood-based assessment of DNA methylation have controlled for cell mixture using complete-blood cell count (CBC) measurements [74,75], such measurements are not capable of distinguishing between different lymphocyte subtypes and may be an oversimplification of the complexity and variability in circulating immune cells. Here, we employed a recently developed statistical method for predicting the distribution of the major leukocyte components of whole blood, followed by their inclusion as additional covariates in our methylation association analyses. It should be noted that the cell type predictions obtained using this approach are themselves estimates and therefore subject to uncertainty. Computationally efficient statistical approaches that facilitate the propagation of this uncertainty into locus-specific differential methylation analyses are urgently needed and represent an opportunity for future methodological work.

A limitation of EWAS aimed toward understanding the molecular basis of complex phenotypes over conventional GWAS, is that the methylation sites identified from EWAS may be a consequence of the disease or due to treatment, rather than true biomarkers of disease risk. We attempted to address this limitation by focusing our analysis on pre-treatment EOC cases and through the implementation of a statistical mediation framework that was recently shown to be an effective tool in the analysis of data arising from EWAS [16]. We do however note that in focusing on the identification of candidate methylation mediators of EOC genetic risk, it is possible, and likely, that other potentially “causal” epigenetic marks were missed using our analytical strategy. Along these lines, there is an urgent need for studies involving the investigation of prospectively collected methylation profiles and subsequent risk of EOC, such as that carried out in a recent study of breast cancer risk [76]. It should be acknowledged that, as in all case-control studies, it is not possible to establish causality on the basis of purely retrospective observational data. With this in mind, our findings can be viewed as a basis for hypotheses, providing a starting point for future mechanistic studies and studies focused on their validation in independent study populations.

## Conclusions

Overall, this study contributes to the growing archive of integrative genomics studies by exploring the relationship between genetics and epigenetics as they relate to EOC risk. Our analysis identified 17 CpG/SNP pairs, comprising 13 unique CpGs and 17 unique SNPs, which represent potential methylation-mediated relationships between genotype and EOC risk. These findings provide additional insight into EOC etiology and may serve as novel biomarkers for EOC susceptibility. Future work is needed to independently validate the genotype-methylation markers discovered here and to elucidate their functional role.

## Abbreviations

EWAS: Epigenome-wide association study; EOC: Epithelial ovarian cancer; CTCs: Circulating tumor cells; LD: Linkage disequilibrium; CIT: Causal inference test; DNAm: DNA methylation; PCA: Principal components analysis.

## Competing interests

The authors declare that they have no competing interests.

## Authors’ contributions

DK carried out the statistical analysis and drafted the manuscript. PC, ML, and SA contributed to the preprocessing and quality control of genomic data used in this investigation, as well as the preparation of the manuscript. MC, JC, MB, TS, and JMC provided assistance in the interpretation of the results and helped in the manuscript preparation. BF and EG conceived of the study, and participated in its design and coordination and helped to draft the manuscript. All authors have read and approve the final manuscript.

## Pre-publication history

The pre-publication history for this paper can be accessed here:

http://www.biomedcentral.com/1755-8794/7/8/prepub

## Supplementary Material

Additional file 1: Table S1 P-values based on examining the association between the top 3 principal components and covariate information. Click here for file

Additional file 2: Figure S1 Plot of the first two principal components computed from the raw DNA methylation data. Black points indicate samples from Batch 1 (n = 132) and red points indicate samples from Batch 2 (n = 296). Click here for file

Additional file 3: Table S2 Pyrosequencing methylation assay and correlation with Illumina 27 K methylation beta values. Click here for file

Additional file 4 Table S3 Information for the 1,993 differentially methylated CpGs between EOC cases and controls. Click here for file

Additional file 5: Table S4 Association of covariates with case/control status. Click here for file

Additional file 6: Table S5 Statistically significant CpG/SNP pairs obtained from examining the association between methylation and genotype. Results obtained for non-statistically significant CpG/SNP associations are available from the authors upon request. Click here for file

Additional file 7: Table S6 Information for the 28 SNPs that were significantly associated with EOC case/control status. Click here for file

Additional file 8: Table S7 Information for the 13 CpGs that were identified as potential methylation-mediated relationships between genotype and EOC risk. Click here for file

Additional file 9: Table S8 Information for the 17 SNPs that were identified as potential methylation-mediated relationships between genotype and EOC risk. Click here for file

Additional file 10: Figure S2 LD-plots for the 17 unique SNPs identified in the mediation analysis among (A) EOC cases and (B) controls. Click here for file

Additional file 11: Figure S3 Methylation levels of the 13 unique CpGs, identified as potential mediators of genetic risk for EOC, across the various EOC histologies. HS (high-grade serous), LS (low-grade serous), M (mucinous), E (endometriod), and C (clear cell). Click here for file
