# Supplementary material for: Integrative genomic analysis identifies epigenetic marks that mediate genetic risk for epithelial ovarian cancer
Source: BMC Med Genomics. 2014 Jan 30;7:8. doi: 10.1186/1755-8794-7-8 (PMC3916313; doi:10.1186/1755-8794-7-8)

Figure S1: Plot of the first two principal components computed from the raw DNA methylation data. Black points indicate samples from Batch 1 (n = 132) and red points indicate samples from Batch 2 (n = 296).

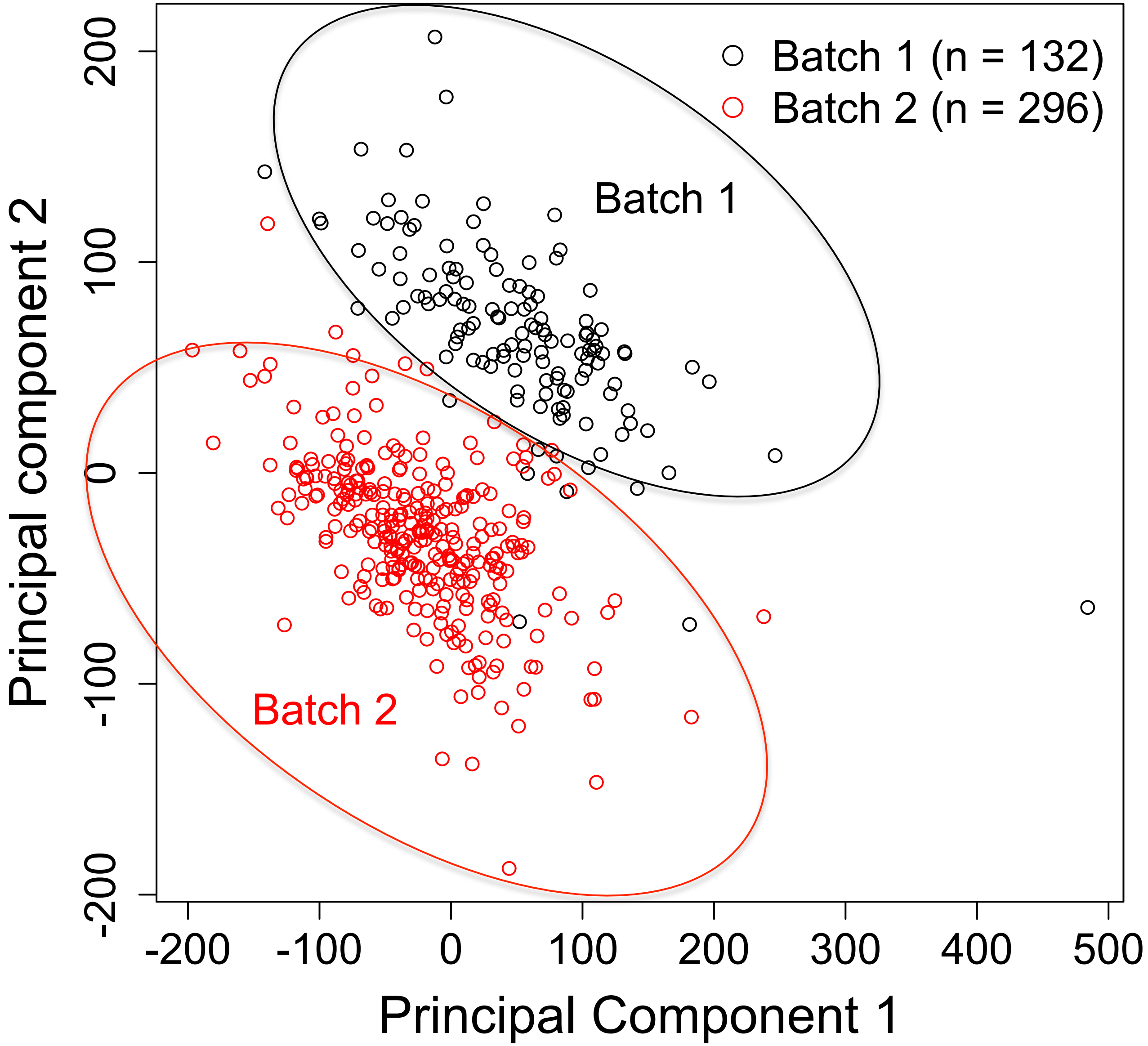

Supplement: Additional file 2: Figure S1 — Plot of the first two principal components computed from the raw DNA methylation data. Black points indicate samples from Batch 1 (n = 132) and red points indicate samples from Batch 2 (n = 296). [file 1755-8794-7-8-S2.pdf]
