# Supplementary material for: Integrative genomic analysis identifies epigenetic marks that mediate genetic risk for epithelial ovarian cancer
Source: BMC Med Genomics. 2014 Jan 30;7:8. doi: 10.1186/1755-8794-7-8 (PMC3916313; doi:10.1186/1755-8794-7-8)
Supplement: Additional file 5: Table S4 — Association of covariates with case/control status. [file 1755-8794-7-8-S5.docx]

| **Table S4: Association of covariates with case/control status** | | | | | | | | |
| --- | --- | --- | --- | --- | --- | --- | --- | --- |
|  | | | | | case (N=214) | control (N=214) | Total (N=428) | p value |
| **Age at Diag/Interview** | | | | |  |  |  | 0.9722 |
| N | | | | | 214 | 214 | 428 |  |
| Mean (SD) | | | | | 62.4 (12.7) | 62.5 (12.7) | 62.4 (12.7) |  |
| Median | | | | | 64.0 | 64.0 | 64.0 |  |
| Q1, Q3 | | | | | 52.0, 72.0 | 52.0, 72.0 | 52.0, 72.0 |  |
| Range | | | | | (28.0-91.0) | (27.0-89.0) | (27.0-91.0) |  |
|  | | | | |  |  |  |  |
| **Ever Used OC** | | | | |  |  |  | 0.2905 |
| Missing | | | | | 5 | 12 | 17 |  |
| yes | | | | | 105 (50.2%) | 112 (55.4%) | 217 (52.8%) |  |
| no | | | | | 104 (49.8%) | 90 (44.6%) | 194 (47.2%) |  |
|  | | | | |  |  |  |  |
| **Weight 1 year ago (kg)** | | | | |  |  |  | 0.3399 |
| N | | | | | 207 | 202 | 409 |  |
| Mean (SD) | | | | | 74.6 (15.9) | 72.5 (14.4) | 73.6 (15.2) |  |
| Median | | | | | 71.7 | 70.3 | 70.3 |  |
| Q1, Q3 | | | | | 63.5, 81.6 | 62.6, 81.6 | 63.5, 81.6 |  |
| Range | | | | | (47.6-129.3) | (46.7-117.9) | (46.7-129.3) |  |
|  | | | | |  |  |  |  |
| **Adult height (m)** | | | | |  |  |  | 0.2482 |
| N | | | | | 210 | 204 | 414 |  |
| Mean (SD) | | | | | 1.6 (0.1) | 1.6 (0.1) | 1.6 (0.1) |  |
| Median | | | | | 1.6 | 1.7 | 1.7 |  |
| Q1, Q3 | | | | | 1.6, 1.7 | 1.6, 1.7 | 1.6, 1.7 |  |
| Range | | | | | (1.5-1.8) | (1.2-1.9) | (1.2-1.9) |  |
|  | | | | |  |  |  |  |
| **Body Mass Index** | | | | |  |  |  | 0.1834 |
| N | | | | | 207 | 202 | 409 |  |
| Mean (SD) | | | | | 27.8 (6.0) | 26.8 (5.2) | 27.3 (5.7) |  |
| Median | | | | | 26.1 | 25.8 | 26.1 |  |
| Q1, Q3 | | | | | 23.4, 30.9 | 23.2, 29.4 | 23.3, 29.9 |  |
| Range | | | | | (18.1-49.0) | (18.5-49.5) | (18.1-49.5) |  |
|  | | | | |  |  |  |  |
| **pregever** | | | | |  |  |  | 0.4057 |
| Missing | | | | | 4 | 10 | 14 |  |
| yes | | | | | 179 (85.2%) | 179 (87.7%) | 358 (86.5%) |  |
| no | | | | | 31 (14.8%) | 24 (11.8%) | 55 (13.3%) |  |
| DK | | | | | 0 (0.0%) | 1 (0.5%) | 1 (0.2%) |  |
|  | | | | |  |  |  |  |
| **Cat. # live births** | | | | |  |  |  | 0.3596 |
| Missing | | | | | 4 | 11 | 15 |  |
| Nulliparous | | | | | 38 (18.1%) | 28 (13.8%) | 66 (16.0%) |  |
| 1-2 | | | | | 71 (33.8%) | 65 (32.0%) | 136 (32.9%) |  |
| 3 or more | | | | | 101 (48.1%) | 110 (54.2%) | 211 (51.1%) |  |
|  | | | | |  |  |  |  |
| **Parity/Age first birth combo** | | | | |  |  |  | 0.0675 |
| Missing | | | | | 4 | 12 | 16 |  |
| Nulliparous | | | | | 38 (18.1%) | 28 (13.9%) | 66 (16.0%) |  |
| 1-2,<=20 yrs | | | | | 10 (4.8%) | 11 (5.4%) | 21 (5.1%) |  |
| 1-2,>20 yrs | | | | | 61 (29.0%) | 54 (26.7%) | 115 (27.9%) |  |
| 3+,<=20 yrs | | | | | 45 (21.4%) | 30 (14.9%) | 75 (18.2%) |  |
| 3+,>20 yrs | | | | | 56 (26.7%) | 79 (39.1%) | 135 (32.8%) |  |
|  | | | | |  |  |  |  |
| **Endometriosis** | | | | |  |  |  | 0.5691 |
| Missing | | | | | 98 | 17 | 115 |  |
| yes | | | | | 9 (7.8%) | 12 (6.1%) | 21 (6.7%) |  |
| no | | | | | 107 (92.2%) | 185 (93.9%) | 292 (93.3%) |  |
|  | | | | |  |  |  |  |
| **1st Deg. w/ OVCA/BRCA** | | | | |  |  |  | 0.3012 |
| yes | | | | | 32 (15.0%) | 40 (18.7%) | 72 (16.8%) |  |
| no | | | | | 182 (85.0%) | 174 (81.3%) | 356 (83.2%) |  |
|  | | | | |  |  |  |  |
| **1st Deg. w/ OVCA** | | | | |  |  |  | 0.6103 |
| yes | | | | | 9 (4.2%) | 7 (3.3%) | 16 (3.7%) |  |
| no | | | | | 205 (95.8%) | 207 (96.7%) | 412 (96.3%) |  |
|  | | | | |  |  |  |  |
| **1/2nd Deg. w/ OVCA/BRCA** | | | | |  |  |  | 0.2494 |
| Missing | | | | | 4 | 10 | 14 |  |
| No | | | | | 124 (59.0%) | 109 (53.4%) | 233 (56.3%) |  |
| Yes | | | | | 86 (41.0%) | 95 (46.6%) | 181 (43.7%) |  |
|  | | | | |  |  |  |  |
| **1/2nd Deg. w/ OVCA** | | | | |  |  |  | 0.1771 |
| Missing | | | | | 4 | 10 | 14 |  |
| No | | | | | 184 (87.6%) | 187 (91.7%) | 371 (89.6%) |  |
| Yes | | | | | 26 (12.4%) | 17 (8.3%) | 43 (10.4%) |  |
|  | | | | |  |  |  |  |
| **Physical activity** | | | | |  |  |  | 0.0277 |
| Missing | | | | | 95 | 12 | 107 |  |
| 4 or more times a week | | | | | 40 (33.6%) | 45 (22.3%) | 85 (26.5%) |  |
| 2 to 4 times a week | | | | | 19 (16.0%) | 53 (26.2%) | 72 (22.4%) |  |
| 1 or less times a week | | | | | 60 (50.4%) | 104 (51.5%) | 164 (51.1%) |  |
|  | | | | |  |  |  |  |
| **Alcohol** | | | | |  |  |  | 0.0026 |
| Missing | | | | | 94 | 12 | 106 |  |
| Never | | | | | 38 (31.7%) | 30 (14.9%) | 68 (21.1%) |  |
| Less than 1 drink per week | | | | | 49 (40.8%) | 90 (44.6%) | 139 (43.2%) |  |
| 1-6 drinks per week | | | | | 21 (17.5%) | 57 (28.2%) | 78 (24.2%) |  |
| 1 drink or more per day | | | | | 12 (10.0%) | 25 (12.4%) | 37 (11.5%) |  |
|  | | | | |  |  |  |  |
| **Current alcohol use** | | | | |  |  |  | <0.0001 |
| Missing | | | | | 18 | 12 | 30 |  |
| Never | | | | | 73 (37.2%) | 30 (14.9%) | 103 (25.9%) |  |
| Current | | | | | 98 (50.0%) | 132 (65.3%) | 230 (57.8%) |  |
| Former | | | | | 25 (12.8%) | 40 (19.8%) | 65 (16.3%) |  |
|  | | | | |  |  |  |  |
| **Multivitamin use** | | | | |  |  |  | 0.6643 |
| Missing | | | | | 93 | 15 | 108 |  |
| No | | | | | 43 (35.5%) | 66 (33.2%) | 109 (34.1%) |  |
| Yes | | | | | 78 (64.5%) | 133 (66.8%) | 211 (65.9%) |  |
|  | | | | |  |  |  |  |
| **enrollyear** | | | | |  |  |  | <0.0001 |
| 1999 | | | | | 3 (1.4%) | 0 (0.0%) | 3 (0.7%) |  |
| 2000 | | | | | 25 (11.7%) | 0 (0.0%) | 25 (5.8%) |  |
| 2001 | | | | | 17 (7.9%) | 0 (0.0%) | 17 (4.0%) |  |
| 2002 | | | | | 46 (21.5%) | 91 (42.5%) | 137 (32.0%) |  |
| 2003 | | | | | 18 (8.4%) | 28 (13.1%) | 46 (10.7%) |  |
| 2004 | | | | | 25 (11.7%) | 49 (22.9%) | 74 (17.3%) |  |
| 2005 | | | | | 30 (14.0%) | 18 (8.4%) | 48 (11.2%) |  |
| 2006 | | | | | 23 (10.7%) | 18 (8.4%) | 41 (9.6%) |  |
| 2007 | | | | | 27 (12.6%) | 10 (4.7%) | 37 (8.6%) |  |
|  | | | | |  |  |  |  |
| **enrollyearc** | | | | |  |  |  | 0.0007 |
| 1999-2002 | | | | | 91 (42.5%) | 91 (42.5%) | 182 (42.5%) |  |
| 2003 | | | | | 18 (8.4%) | 28 (13.1%) | 46 (10.7%) |  |
| 2004 | | | | | 25 (11.7%) | 49 (22.9%) | 74 (17.3%) |  |
| 2005 | | | | | 30 (14.0%) | 18 (8.4%) | 48 (11.2%) |  |
| 2006+ | | | | | 50 (23.4%) | 28 (13.1%) | 78 (18.2%) |  |
|  | | | | |  |  |  |  |
| **state2** | | | | |  |  |  | 0.0240 |
| MN | | | | | 111 (51.9%) | 129 (60.3%) | 240 (56.1%) |  |
| IA | | | | | 30 (14.0%) | 36 (16.8%) | 66 (15.4%) |  |
| WI | | | | | 30 (14.0%) | 26 (12.1%) | 56 (13.1%) |  |
| IL | | | | | 8 (3.7%) | 9 (4.2%) | 17 (4.0%) |  |
| ND/SD | | | | | 35 (16.4%) | 14 (6.5%) | 49 (11.4%) |  |
|  | | | | |  |  |  |  |
| **mn_state** | | | | |  |  |  | 0.0796 |
| Other | | | | | 103 (48.1%) | 85 (39.7%) | 188 (43.9%) |  |
| MN | | | | | 111 (51.9%) | 129 (60.3%) | 240 (56.1%) |  |
|  | | | | |  |  |  |  |
| **Cat. age first live birth** | | | | |  |  |  | 0.1093 |
| Missing | | | | | 4 | 12 | 16 |  |
| Nulliparous | | | | | 38 (18.1%) | 28 (13.9%) | 66 (16.0%) |  |
| <=20 yrs | | | | | 55 (26.2%) | 41 (20.3%) | 96 (23.3%) |  |
| >20 yrs | | | | | 117 (55.7%) | 133 (65.8%) | 250 (60.7%) |  |
|  | | | | |  |  |  |  |
| **Menopause status** | | | | |  |  |  | 0.8932 |
| Missing | | | | | 6 | 10 | 16 |  |
| pre/peri | | | | | 46 (22.1%) | 44 (21.6%) | 90 (21.8%) |  |
| post | | | | | 162 (77.9%) | 160 (78.4%) | 322 (78.2%) |  |
|  | | | | |  |  |  |  |
| **Cat. Age Menarche** | | | | |  |  |  | 0.2816 |
| Missing | | | | | 47 | 16 | 63 |  |
| 11 | | | | | 27 (16.2%) | 30 (15.2%) | 57 (15.6%) |  |
| 12 | | | | | 45 (26.9%) | 42 (21.2%) | 87 (23.8%) |  |
| 13 | | | | | 44 (26.3%) | 70 (35.4%) | 114 (31.2%) |  |
| 14 | | | | | 51 (30.5%) | 56 (28.3%) | 107 (29.3%) |  |
|  | | | | |  |  |  |  |
| **HRT Ever** | | | | |  |  |  | 0.1139 |
| Missing | | | | | 4 | 10 | 14 |  |
| yes | | | | | 81 (38.6%) | 98 (48.0%) | 179 (43.2%) |  |
| no | | | | | 128 (61.0%) | 104 (51.0%) | 232 (56.0%) |  |
| DK | | | | | 1 (0.5%) | 2 (1.0%) | 3 (0.7%) |  |
|  | | | | |  |  |  |  |
| **Cat. months HRT use** | | | | |  |  |  | 0.2938 |
| Missing | | | | | 7 | 24 | 31 |  |
| Never | | | | | 128 (61.8%) | 104 (54.7%) | 232 (58.4%) |  |
| 1-60 Months | | | | | 32 (15.5%) | 39 (20.5%) | 71 (17.9%) |  |
| 60+ Months | | | | | 47 (22.7%) | 47 (24.7%) | 94 (23.7%) |  |
|  | | | | |  |  |  |  |
| **Ever Used OC** | | | | |  |  |  | 0.2905 |
| Missing | | | | | 5 | 12 | 17 |  |
| yes | | | | | 105 (50.2%) | 112 (55.4%) | 217 (52.8%) |  |
| no | | | | | 104 (49.8%) | 90 (44.6%) | 194 (47.2%) |  |
|  | | | | |  |  |  |  |
| **Total months of oral contraceptive use** | | | | |  |  |  | 0.0182 |
| N | | | | | 201 | 198 | 399 |  |
| Mean (SD) | | | | | 32.7 (56.7) | 53.7 (78.1) | 43.1 (68.9) |  |
| Median | | | | | 0.0 | 12.0 | 3.0 |  |
| Q1, Q3 | | | | | 0.0, 48.0 | 0.0, 84.0 | 0.0, 60.0 |  |
| Range | | | | | (0.0-300.0) | (0.0-480.0) | (0.0-480.0) |  |
|  | | | | |  |  |  |  |
| **Cat. months OC use** | | | | |  |  |  | 0.0028 |
| Missing | | | | | 13 | 16 | 29 |  |
| Never | | | | | 104 (51.7%) | 90 (45.5%) | 194 (48.6%) |  |
| 1-48 Months | | | | | 50 (24.9%) | 32 (16.2%) | 82 (20.6%) |  |
| 48+ Months | | | | | 47 (23.4%) | 76 (38.4%) | 123 (30.8%) |  |
|  | | | | |  |  |  |  |
| **Used Combination Estrogen/Progestin HRT** | | | | |  |  |  | 0.8551 |
| Missing | | | | | 94 | 12 | 106 |  |
| yes | | | | | 38 (31.7%) | 62 (30.7%) | 100 (31.1%) |  |
| no | | | | | 82 (68.3%) | 140 (69.3%) | 222 (68.9%) |  |
|  | | | | |  |  |  |  |
|  | | | | |  |  |  |  |
| **Highest Education achieved (<HS, HS, Beyond HS)** | | | | |  |  |  | 0.0004 |
| Missing | | | | | 10 | 10 | 20 |  |
| No Diploma | | | | | 11 (5.4%) | 8 (3.9%) | 19 (4.7%) |  |
| High School Diploma | | | | | 82 (40.2%) | 47 (23.0%) | 129 (31.6%) |  |
| Post HS Education | | | | | 111 (54.4%) | 149 (73.0%) | 260 (63.7%) |  |
|  | | | | |  |  |  |  |
| **Took Fertility Meds** | | | | |  |  |  | 0.5707 |
| Missing | | | | | 102 | 178 | 280 |  |
| No | | | | | 103 (92.0%) | 32 (88.9%) | 135 (91.2%) |  |
| Yes | | | | | 9 (8.0%) | 4 (11.1%) | 13 (8.8%) |  |
|  | | | | |  |  |  |  |
| **Cat. packyrs 0,4,4+** | | | | |  |  |  | 0.2659 |
| Missing | | | | | 14 | 21 | 35 |  |
| none | | | | | 130 (65.0%) | 132 (68.4%) | 262 (66.7%) |  |
| <=4 | | | | | 14 (7.0%) | 19 (9.8%) | 33 (8.4%) |  |
| >4 | | | | | 56 (28.0%) | 42 (21.8%) | 98 (24.9%) |  |
|  | | | | |  |  |  |  |
| **Cat. packyrs 0,20,20+** | | | | |  |  |  | 0.0205 |
| Missing | | | | | 14 | 21 | 35 |  |
| none | | | | | 130 (65.0%) | 132 (68.4%) | 262 (66.7%) |  |
| <=20 | | | | | 33 (16.5%) | 43 (22.3%) | 76 (19.3%) |  |
| >20 | | | | | 37 (18.5%) | 18 (9.3%) | 55 (14.0%) |  |
|  | | | | |  |  |  |  |
|  | | | | |  |  |  |  |
| **Ever/never smoke** | | | | |  |  |  | 0.5515 |
| Missing | | | | | 5 | 11 | 16 |  |
| Never | | | | | 130 (62.2%) | 132 (65.0%) | 262 (63.6%) |  |
| Ever | | | | | 79 (37.8%) | 71 (35.0%) | 150 (36.4%) |  |
|  | | | | |  |  |  |  |
| **Never/former/current smoke** | | | | |  |  |  | 0.2218 |
| Missing | | | | | 9 | 11 | 20 |  |
| Never | | | | | 130 (63.4%) | 132 (65.0%) | 262 (64.2%) |  |
| Former | | | | | 56 (27.3%) | 61 (30.0%) | 117 (28.7%) |  |
| Current | | | | | 19 (9.3%) | 10 (4.9%) | 29 (7.1%) |  |
|  | | | | |  |  |  |  |
| **Current smoke** | | | | |  |  |  | 0.0879 |
| Missing | | | | | 9 | 11 | 20 |  |
| No | | | | | 186 (90.7%) | 193 (95.1%) | 379 (92.9%) |  |
| Yes | | | | | 19 (9.3%) | 10 (4.9%) | 29 (7.1%) |  |
|  |  |  |  |  |  |  |  |  |
|  | | | | |  |  |  |  |
|  | | | | |  |  |  |  |
|  | | | | |  |  |  |  |
|  | | | | |  |  |  |  |
|  | | | | |  |  |  |  |
|  | | | | |  |  |  |  |
|  | | | | |  |  |  |  |
|  | | | | |  |  |  |  |
|  | | | | | | | | |
