# Supplementary material for: Integrative genomic analysis identifies epigenetic marks that mediate genetic risk for epithelial ovarian cancer
Source: BMC Med Genomics. 2014 Jan 30;7:8. doi: 10.1186/1755-8794-7-8 (PMC3916313; doi:10.1186/1755-8794-7-8)

Figure S2: LD-plots for the 17 unique SNPs identified in the mediation analysis among (A) EOC cases and (B) controls.

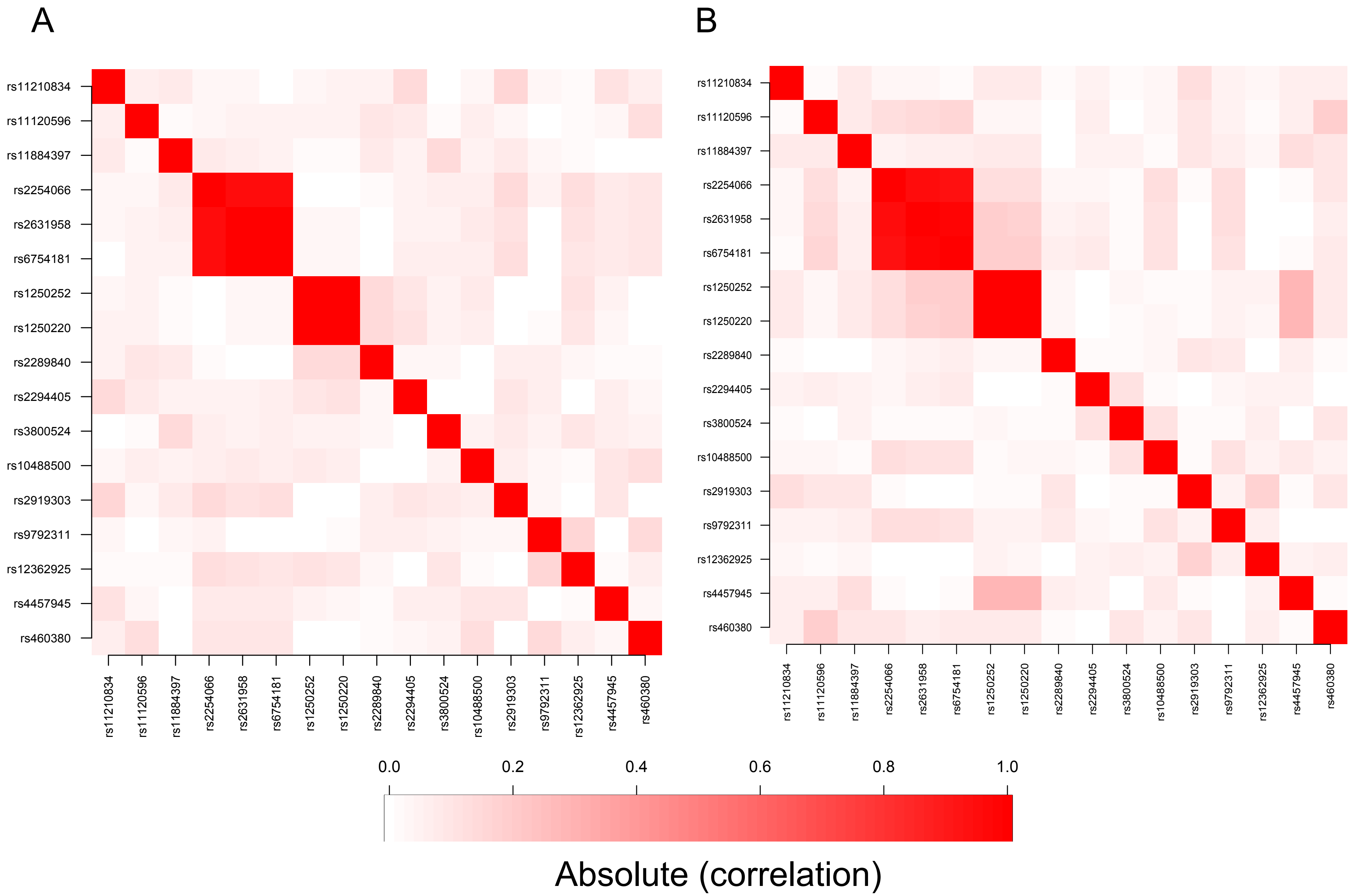

Supplement: Additional file 10: Figure S2 — LD-plots for the 17 unique SNPs identified in the mediation analysis among (A) EOC cases and (B) controls. [file 1755-8794-7-8-S10.pdf]
