# Supplementary material for: Integrative genomic analysis identifies epigenetic marks that mediate genetic risk for epithelial ovarian cancer
Source: BMC Med Genomics. 2014 Jan 30;7:8. doi: 10.1186/1755-8794-7-8 (PMC3916313; doi:10.1186/1755-8794-7-8)

Figure S3: Methylation levels of the 13 unique CpGs, identified as potential mediators of genetic risk for EOC, across the various EOC histologies. HS (high-grade serous), LS (low-grade serous), M (mucinous), E (endometriod), and C (clear cell).

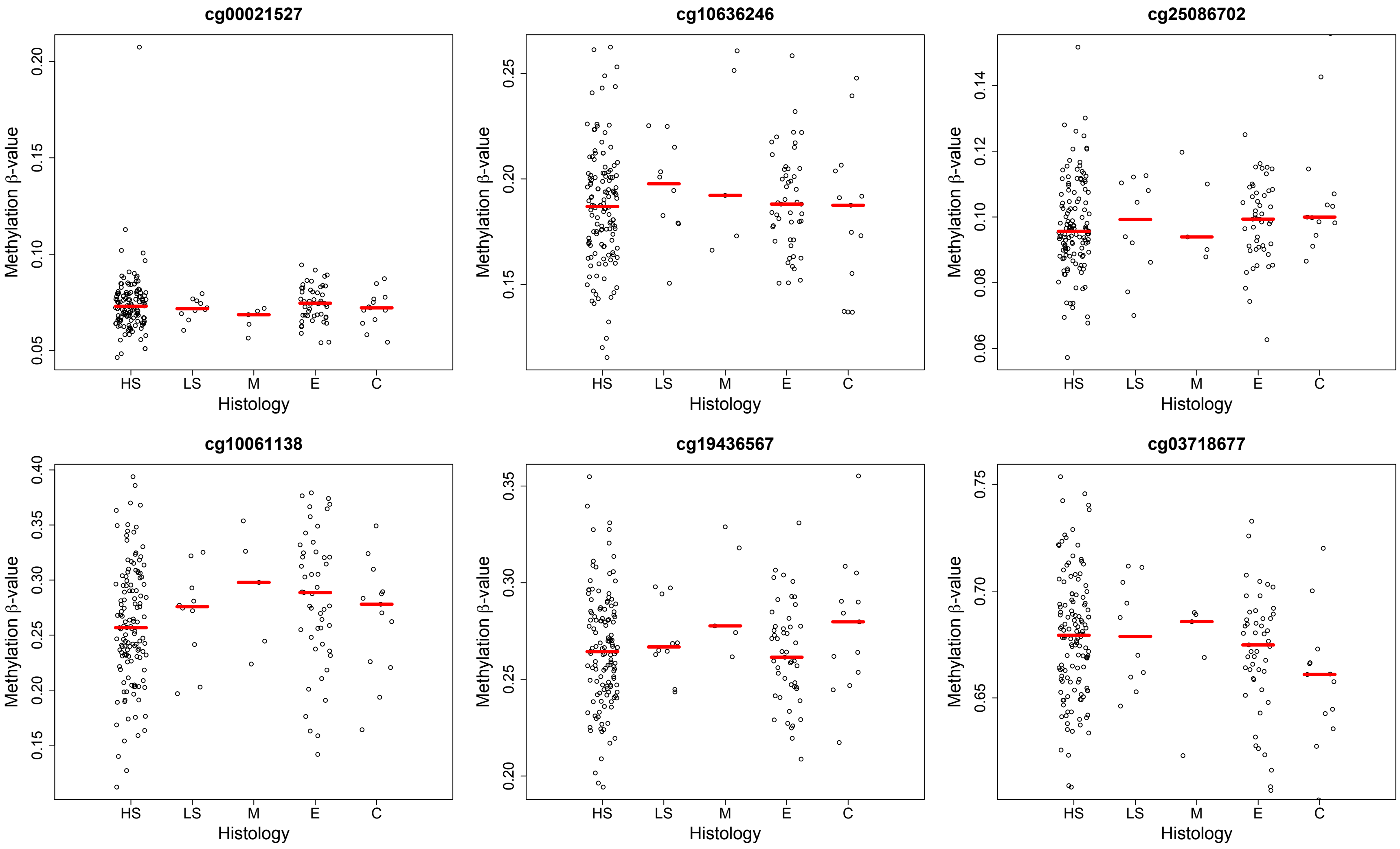

Figure S2 (cont.)

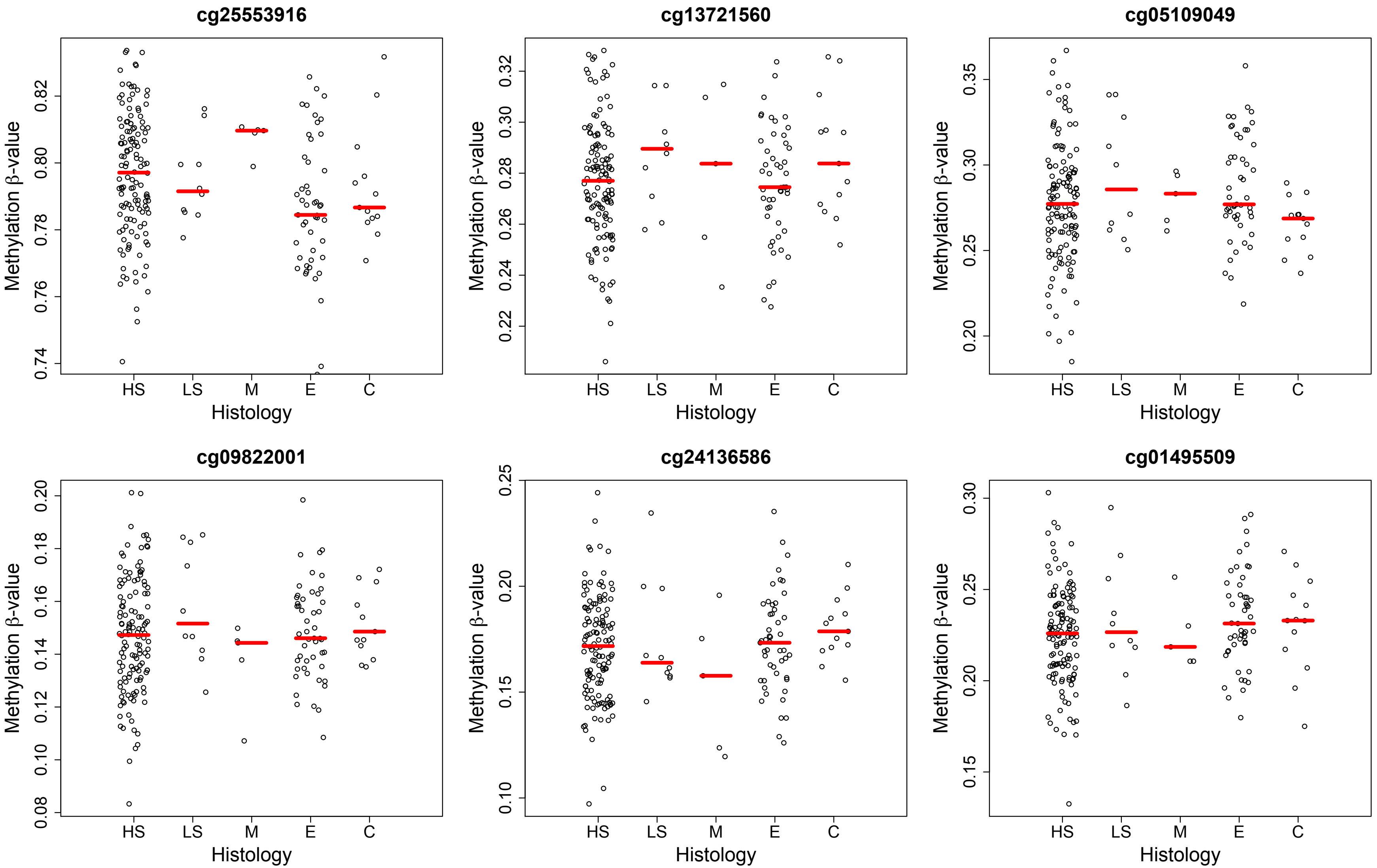

Figure S2 (cont.)

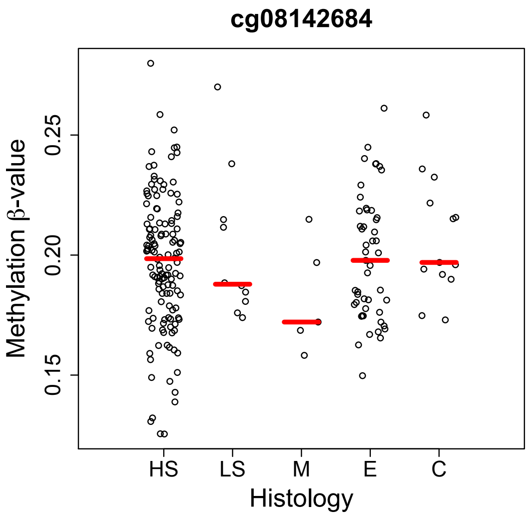

Supplement: Additional file 11: Figure S3 — Methylation levels of the 13 unique CpGs, identified as potential mediators of genetic risk for EOC, across the various EOC histologies. HS (high-grade serous), LS (low-grade serous), M (mucinous), E (endometriod), and C (clear cell). [file 1755-8794-7-8-S11.pdf]
